# Relationship between sleep pattern dysfunction with psychological stress, anxiety and depression among pregnant women

**DOI:** 10.12669/pjms.41.1.9413

**Published:** 2025-01

**Authors:** Afshan Tariq, Waqas Ahmed Farooqui, Shazia Jabbar Khan, Kashif Shafique

**Affiliations:** 1 Afshan Tariq, MSBE Student, Dow University of Health Sciences, Karachi, Pakistan; 2 Waqas Ahmed Farooqui Associate Professor, School of Public Health, Dow University of Health Sciences, Karachi, Pakistan; 3 Shazia Jabbar Khan Associate Professor, Department of Obstetrics and Gynecology, Dow University Hospital, Dow University of Health Sciences. Karachi, Pakistan. Dow University of Health Sciences, Karachi, Pakistan; 4 Kashif Shafique Professor of Public Health & Principal, School of Public Health, Dow University of Health Sciences, Karachi, Pakistan

**Keywords:** Depression, Stress, Anxiety, Pregnancy

## Abstract

**Objective:**

To determine the relationship between sleep pattern dysfunction with stress, anxiety and depression among pregnant women in a tertiary care hospital.

**Method:**

An analytical cross-sectional study was conducted at Dr. Ruth K. M. Pfau at Civil Hospital, Karachi from December, 2021 till January, 2022 in which total three hundred pregnant women were included. The association between maternal psychiatric symptoms with the sleep pattern was explored using the Chi- square test. The multivariable logistic regression technique was used to estimate adjusted odds ratios (ORs) and to estimate the association of sleep pattern dysfunction with psychiatric illnesses.

**Results:**

Sleep disorder was found in 49.3% of the pregnant women. Among these women, 54.0% had antepartum depression, 31.3% had stress and 44% reported anxiety. Women with sleep pattern dysfunction were nine times more likely to develop antepartum depression as compared to women without sleep pattern dysfunction (OR: 9.25, 95% CI: 5.45-15.70). Similarly, stress was five times more likely to develop in women with sleep pattern dysfunction (OR: 5.06, 95% CI: 2.92-8.76). Furthermore, Anxiety is also four times likely to develop in women with sleep pattern dysfunction (OR: 4.76, 95% CI: 2.91-7.80) compared to women without any sleep disturbances.

**Conclusions:**

During pregnancy, sleep disturbances were related to the increased risk of developing psychiatric illnesses. It was found that women with disturbed sleep patterns had high rates of antepartum depression followed by stress and anxiety as compared to women without disturbed sleep patterns.

## INTRODUCTION

Sleep is an essential component of good health required for repair and relieving functions. It is essential for pregnant women for the transfer of energy to fetoplacental unit.^1^ According to the report of the National Sleep Foundation, nearly 80% of women, at some point, during pregnancy go through poor and disturbed sleep. The sleep disturbances are secondary to physiological and hormonal changes taking place during pregnancy.^2^ These changes in turn may interfere with sleep architecture, continuity and may worsen any preexisting sleep conditions as well as causing problems during and after pregnancy.

The relationship between quality of sleep and depression in pregnancy is found to be significant and clinically related as the poorer the sleep the higher depressive and anxiety symptoms severity scores were calculated.^3^ The WHO estimated that 10-16% of women during pregnancy and 13-20% of women in the postpartum period experiences mental illnesses with the major contribution is of depression.^4^ This percentage is even high in developing countries with low resources, reaching up to 15.6%.^5^ The sleep disturbances affect maternal quality of life causing pregnancy related complications like pre-eclampsia, gestational diabetes and is also associated with adverse pregnancy outcomes like preterm birth, IUGR and still birth.^6^

According to a meta-analysis done to assess poor sleep quality during pregnancy there is an impact of self-reported poor sleep on the development of postpartum depression, anxiety and stress. Insomnia is frequently encountered in pregnant women. A study reported that throughout pregnancy 45.7% of women undergo a significant reduction in sleep quality and experience poor sleep.^7^ In the first trimester, over 40% of pregnant women undergo insomnia, which increases up to 60% to 72.6% by the third trimester.^8^,^9^

Socio-demographic factors can influence mental health and are mostly accountable for severe mental health issues including lack of family support, partner violence, low economic status, and obstetrics factors like unplanned pregnancy. Despite the fact that the rates of maternal anxiety and depression are higher in low and middle-income countries (LMICs) most studies on psychiatric disorders among pregnant women were conducted in high-income countries.^10^ Women from low-income backgrounds are more likely to face psychological problems as they worry about the future due to the expected increase in family size with limited income resources.^2^ Information regarding the association between sleep disturbance and psychiatric disorders among pregnant women is limited.

This Calls for a dire need of screening tools that can easily identify the women who are at risk for antenatal depression & postpartum psychosis. PSQI tool appears to be an appropriate measure to evaluate sleep quality.^11^ similarly the DASS-21 tool (Depression, Anxiety and Stress Scale) using socio demographic characteristics can be used in research work and practices.^12^

A study conducted in Pakistan, using PSQI tool found that 73.7% of pregnant women experience poor sleep terms of frequency and quality.^9^ Another study in Pakistan reported the levels of depression categorized as normal (43.6%), borderline (24.6%) and depressed (31.8%) in pregnant women.^13^ A systemic review conducted in Pakistan revealed that the combined prevalence of antenatal depression stood at 37% whereas postnatal depression was found to be 30%.^14^ In a research effort in Hyderabad, Sindh, depression frequency during pregnancy was observed at 18% using the Aga Khan University Anxiety Depression Scale (AKUADS) in the latter part of the 2^nd^ trimester. In a separate study in Punjab, the prevalence of antenatal depression was determined to be 25%. Additionally, a descriptive cross-sectional study in District Chitral predicted a 34% prevalence of depression among a sample of 340 pregnant women.^15^

During pregnancy sleep disruption and inadequate sleep is a frequent complaint of women. The present study is therefore conducted in a tertiary care hospital to screen and identify women with sleep reactivity (individuals with sleep system sensitive to stress) and its association with psychiatric illnesses. This will help in identifying at risk women at an early stage so that prevention strategies and timely interventions can be enhanced to improve maternal and perinatal outcome. Interventions and early screening for both high sleep reactivity and psychiatric illness should be included as a part of antenatal care in OPDs.

## METHODS

The analytical, cross-sectional study was conducted in the antenatal OPD of obstetrics and gynecology, Dr. Ruth K. M. Pfau Civil Hospital Karachi, which is a tertiary care hospital from 16^th^ Dec.’21 till 31^st^ Jan.’22. Total 300 patients were included in the study by calculating the sample size using association between stress related sleep disturbances with psychiatric illnesses during pregnancy as 33.2% of women.^16^ An informed written consent was obtained from all the participants. We assessed the mentioned psychiatric disorders (Depression, Anxiety and Stress) in the participants by using the following tools:

- Pittsburgh sleep quality index (PSQI).
- Depression, Anxiety and Stress Scale (DASS-21).

### Study participants:

The pregnant women were selected with no co morbidity between the ages of 18-45 years and of any parity. We excluded women with a past or current self-reported history of any psychiatric illness, women with any high-risk factor were also excluded such as pregnancies at extreme of ages (<15 or >45 years), multiple pregnancy, history of adverse pregnancy outcome, pregnancy with medical disorders or with sexually transmitted diseases. e.g. human immunodeficiency virus/acquired immunodeficiency syndrome, human papillomavirus.

### Ethical consideration:

Ethical approval was sought from the Institutional Review Board (Appendix # VII, Ref # IRB-2198/DUHS/Approval/2021/527, dated: 24^th^ September 2021), DUHS.

### Statistical Analysis:

We examined Socio-demographic and some of the features related to pregnancy by using mean ± standard deviation or median (interquartile range - IQR) for quantitative variables (age, gestational age, Hb, MCV, MCH, MCHC), etc. numbers and percentages for categorical variables (ethnicity, employment status), etc.

For the association between maternal psychiatric symptoms with pattern of sleep, the chi-square test was used. The univariable and multivariable logistic regression technique was used to estimate unadjusted and adjusted odds ratios (ORs) respectively with 95% confidence interval (CI). Binary logistic regression was used to carry out the univariate and multivariable analysis. The cut-off p-value of ≤0.25 and ≤ 0.05 was considered significant for univariate and multivariable regression respectively. Those factors which had p-value ≤0.25 in univariate was taken for further multivariable logistic regression analysis. In multivariable logistic regression a p-value less than or equal to 0.05 considered statistically significant. All statistical calculations were carried out using IBM SPSS version 22.

## RESULTS

The Socio demographic and pregnancy characteristics of three hundred participants were evaluated. The median (interquartile range (IQR)) age for women was 25.0 (21.2 - 29.0) years with mean gestational age 26.0 weeks’ (21.0 - 31.0) weeks. Regarding ethnicity 27% belonged to Urdu speaking community, 70% had less than six years of education and 81.7% were unemployed. Difficulty in accessing basic food was reported by 31% of women. Women whose husbands were employed were 80.3 % and 60% reported monthly household income less than Rs. 25,000. Women with more than one child were 56%, 99.7 % had planned pregnancy and 40% were found to be overweight. Tobacco uses before or during pregnancy was observed in 39.3% of women. The frequency of sleep disturbance was reported by 49.3%, depression, stress and anxiety were found in 54 %, 31.3 %, 44 % respectively, (Table-I).

**Table-I:** Descriptive Statistics of Baseline Characteristics.

Basic characteristics	n= 300 (%)	Pregnancy characteristics	n= 300 (%)
Age ^a^	25.0 (21.2 - 29.0)	Parity	
Ethnicity		Nulliparous	132 (44.0)
Sindhi	33 (11.0)	Multiparous	168 (56.0)
Punjabi	36 (12.0)	Plan. Preg.	299 (99.7)
Balochi	32 (10.7)	Tob. Use.	118 (39.3)
Pushto	69 (23.0)	Gestational age ^a^	26.0 (21.0 – 31.0)
Urdu	81 (27.0)	CBC Components	
Others	49 (16.3)	Hb^b^	10.64 + 1.49
Employment	55 (18.3)	MCV ^a^	83.0 (76.2 – 87.0)
Husb. Empl.	241 (80.3)	MCH^a^	26.0 (22.4 – 28.9)
Edu. (Years)		MCHC^a^	32.7 (31.1 – 34.5)
< 6	210 (70.0)	Platelets^b^	328.09 + 69.60
7– 12	84 (28.0)	Weight^b^	63.53+ 9.06
>12	6 (2)	Height^b^	154.23+ 6.08
***Diff. access basic food***		***BMI (kg/m^2^)***	
Hard	93 (31.3)	Underweight (< 18.5)	3 (1.0)
Not hard	207 (69.7)	Normal BMI (18.5 – 24.9)	104 (34.7)
Income(‘000)		Overweight (25 - 29.9)	120 (40.0)
< 25	180 (60.0)	Obese (> 30)	73 (24.3)
> 25	111 (37.0)	Depression	162 (54.0)
> 50	9 (3.0)	Anxiety	132 (44.0)
>75	0 (0)	Stress	94 (31.3)

a Median (Inter quartile range),

b Mean ± Standard deviation (SD), Husb. Empl: Husband employment status, Edu.:Years of education, Diff. asses basic food: Difficulty accessing basic food, Plan. Preg.: Planned pregnancy, Tob.: Tobacco, BMI: Body mass index, CBC: Complete blood count components, Hb: Hemoglobin, Ethnicity (others) = (included all languages other than Sindhi, Punjabi, Balochi, Pushto, Urdu).

In our study Antepartum depression, stress and anxiety was found in 79.2%, 47.9% and 62.8% of women with sleep disturbances respectively. In all these conditions calculated P value was significant as <0.001. Disturbed sleep was significantly related to each of the psychiatric illnesses that we studied in our study, (Table-II).

**Table-II:** Association of sleep pattern with psychiatric illnesses.

	With Sleep disturbance	Without Sleep disturbance	

Parameters	(n=148)	(n=152)	P-value^∞^
***Depression***			
No	31 (20.8)	107 (70.9)	<0.001
Yes	117 (79.2)	45 (29.6)	
***Stress***			
No	77 (52.3)	129 (84.8)	<0.001
Yes	71 (47.9)	23 (15.2)	
***Anxiety***			
No	55 (37.6)	113 (74.2)	<0.001
Yes	93 (62.8)	39 (25.8)	

∞ chi-square test.

It was also found that women who had sleep disturbance were nine times more likely to develop antenatal depression (OR: 9.25, 95% CI: 5.45-15.70). These women were five times more likely to develop stress (OR: 5.06, 95% CI: 2.92-8.76) and four times more likely to develop anxiety as compared to women who didn’t have sleep disturbance. (OR: 4.76, 95% CI: 2.91-7.80), (Table-III).

**Table-III:** Association of sleep pattern with psychiatric illnesses.

	Unadjusted OR (95% CI)	Adjusted OR (95% CI)
***Depression***		
Without sleep pattern dysfunction	1	1
With sleep pattern dysfunction	9.25 (5.45-15.70)	16.02(7.20-35.64)
***Stress***		
Without sleep pattern dysfunction	1	1
With sleep pattern dysfunction	5.06 (2.92-8.76)	5.53 (3.11-9.85)
***Anxiety***		
Without sleep pattern dysfunction	1	1
With sleep pattern dysfunction	4.76 (2.91-7.80)	4.86 (2.89-8.15)

OR: Odds ratio adjusted for employment status, husband employment, difficulty accessing basic food, monthly income, parity, tobacco use.

On multivariable analysis, after adjusting for confounders (employment status, husband employment, difficulty accessing basic food, monthly income, parity, and tobacco use) we found that the women with sleep disturbance had sixteen times (OR: 16.02, 95% CI: 7.20-35.64) increased risk of developing antepartum depression.

## DISCUSSION

The result of this study indicates that sleep disorders is frequently encountered in pregnancy resulting in antepartum depression followed by anxiety and stress as compared to women with normal sleep. In our study disturbed sleep was reported in 49.3% of women similar to studies conducted in Iran reporting sleep disorders in 60% of women during pregnancy^6^ and in Peru reported 33.4% of sleep disturbance among pregnant women. A study by Sanchez et al. reported stress related sleep disorders in 33.2% of women and out of these 32.2% had a generalized anxiety disorder, 24.9% had antepartum depression, 27.6% of women were assessed as high risk for psychosis and 30.9% had post-traumatic stress disorder.^16^ Another study in Italy found high stress-related sleep reactivity 46.7% with greater symptoms of insomnia, high proportions for depression 62%, anxiety 55.1%, and high rates for suicidal thoughts in 17.2%.^17^ A study conducted in Iran also documented a significant relationship between depression and sleep quality with sleep duration and efficacy having an inverse relationship with depression score.^6^

The sleep pattern disturbance in our study was significantly related to each of the psychiatric illnesses (antenatal depression, stress and anxiety) which is comparable to the study done by Gao M et al. where it was found that poor sleep quality was positively associated with antenatal depression, stress and also postpartum depression status.^18^ In the present study, it was found that women who had sleep disturbance were nine times more likely to develop antenatal depression which is in accordance to a study by Asltoghiria M et al. which also stated the risk of depression was increased nine folds among women with sleep disturbances.^19^ Another study done in Chinese women also found the association of sleep quality with depression and anxiety among pregnant women.^20^

The median age in our study was 25.0 years and 56% were multiparous women similar to studies in which mean ages was observed 27.92 years and multiparity was observed in 54% and 49.1% of women respectively.^6^,^16^ The mean gestational age in our study was 26weeks’ whereas studies by Firouzbakht M et al.^6^ and Kızılırmak et al. demonstrated that sleep quality deteriorates with an increase in gestational age.^21^ Body mass index is also found to be directly proportional to the sleep disturbances in our study as sleep problems were encountered more in overweight category with a frequency of 43.9% which is in accordance with the studies where insomnia is observed was in 58.9% women with BMI > 25.^6^,^22^

A study by Chunhua Song et al determined the association of passive smoking with postpartum depression (PPD) in Chinese women. In this study it was reported that passive smoking before and during pregnancy were significantly associated with postpartum depression in Chinese women.^23^ This study findings are comparable to our study where the number of women who used tobacco were higher in the group of women who were depressed as compared to other psychiatric illnesses, we studied in both with and without sleep pattern dysfunction women.

The mother and child health are an important component of public health. Many women may experience a mental health problem either in the antenatal period or first year postpartum. Based on the present study findings screening for sleep disturbances in pregnant women as part of antenatal care is recommended. The aim is to identify women prone to psychiatric illnesses due to high sleep reactivity causing poor sleep quality. It is therefore suggested to take into consideration the risk factors and implement strategies for timely intervention to address these sleep disturbances which will help to reduce anxiety and depression in the antenatal as well as post-natal period.

### Strengths of Study:

The information was collected using validated and structured questionnaires which is the strength of our study also it’s the first study that assessed this kind of relationship in a tertiary care setup among pregnant women in Pakistan.

### Limitations:

Exposure and Outcome measured in our study were based on self-report and cross-sectional. Accordingly, the reported measures of association may be subject to non-systematic recall errors. The results may not be generalized as the study participants belonged to the middle to low-income family.

## CONCLUSIONS

During pregnancy, disturbed sleep patterns is found to be related to the increased risk of psychiatric illnesses. The women with disturbed sleep patterns had high rate of antepartum depression followed by anxiety and stress as compared to women without disturbed sleep patterns.

### Author Contribution:

**AT:** The principal investigator, conception, design, data collection, sample processing, data analysis and interpretation and prepared the m, data analysis and interpretation.

**SJK:** Data collection, critical evaluation and review.

**KS:** Supervised the project, interpretation & critical evaluation.

All authors have approved the final version and are accountable for the integrity of the study.
